# Hepatic sinusoidal obstruction syndrome and short-term application of 6-thioguanine in pediatric acute lymphoblastic leukemia

**DOI:** 10.1038/s41375-021-01203-7

**Published:** 2021-03-13

**Authors:** Martin Stanulla, Elke Schaeffeler, Anja Möricke, Swantje Buchmann, Martin Zimmermann, Svitlana Igel, Kjeld Schmiegelow, Christian Flotho, Hans Hartmann, Sabine Illsinger, Axel Sauerbrey, Stefanie V. Junk, Peter Schütte, Laura Hinze, Melchior Lauten, Simon Modlich, Reinhard Kolb, Claudia Rossig, Georg Schwabe, Astrid K. Gnekow, Gudrun Fleischhack, Paul Gerhard Schlegel, Holger J. Schünemann, Christian P. Kratz, Gunnar Cario, Martin Schrappe, Matthias Schwab

**Affiliations:** 1 Pediatric Hematology and Oncology, Hannover Medical School, Hannover, Germany; 2 Dr. Margarete-Fischer-Bosch Institute of Clinical Pharmacology, Stuttgart, Germany; 3 Cluster of Excellence iFIT (EXC2180) “Image-guided and Functionally Instructed Tumor Therapies”, University of Tübingen, Tübingen, Germany; 4 Department of Pediatrics, University Hospital Schleswig-Holstein, Kiel, Germany; 5 Department of Pediatrics and Adolescent Medicine, University Hospital Rigshospitalet, Copenhagen, Denmark; 6 Department of Pediatric Hematology and Oncology, University Hospital Freiburg, Freiburg, Germany; 7 Department of Pediatric Kidney, Liver, and Metabolic Diseases, Hannover Medical School, Hannover, Germany; 8 Pediatric Clinics, Helios Hospital, Erfurt, Germany; 9 Department of Pediatrics, University Hospital Schleswig-Holstein, Lübeck, Germany; 10 University Children’s Hospital, Oldenburg, Germany; 11 Pediatric Hematology and Oncology, University Children’s Hospital Münster, Münster, Germany; 12 Carl Thiem Hospital, Pediatric Clinics, Cottbus, Germany; 13 Pediatric Clinics, University Hospital Augsburg, Augsburg, Germany; 14 Pediatrics III, Pediatric Hematology and Oncology, University Hospital Essen, Essen, Germany; 15 Pediatric Hematology and Oncology and Stem Cell Transplantation, University Hospital Würzburg, Würzburg, Germany; 16 Departments of Health Research Methods, Evidence, and Impact and of Medicine, McMaster University, Hamilton, ON Canada; 17 Departments of Clinical Pharmacology, and of Pharmacy and Biochemistry, University of Tübingen, Tübingen, Germany

**Keywords:** Acute lymphocytic leukaemia, Chemotherapy

## Abstract

Long-term treatment with 6-thioguanine (6-TG) for pediatric acute lymphoblastic leukemia (ALL) is associated with high rates of hepatic sinusoidal obstruction syndrome (SOS). Nevertheless, current treatment continues to use short-term applications of 6-TG with only sparse information on toxicity. 6-TG is metabolized by thiopurine methyltransferase (TPMT) which underlies clinically relevant genetic polymorphism. We analyzed the association between hepatic SOS reported as a serious adverse event (SAE) and short-term 6-TG application in 3983 pediatric ALL patients treated on trial AIEOP-BFM ALL 2000 (derivation cohort) and defined the role of *TPMT* genotype in this relationship. We identified 17 patients (0.43%) with hepatic SOS, 13 of which with short-term exposure to 6-TG (*P* < 0.0001). Eight of the 13 patients were heterozygous for low-activity *TPMT* variants, resulting in a 22.4-fold (95% confidence interval 7.1–70.7; *P* ≤ 0.0001*)* increased risk of hepatic SOS for heterozygotes in comparison to *TPMT* wild-type patients. Results were supported by independent replication analysis. All patients with hepatic SOS after short-term 6-TG recovered and did not demonstrate residual symptoms. Thus, hepatic SOS is associated with short-term exposure to 6-TG during treatment of pediatric ALL and SOS risk is increased for patients with low-activity *TPMT* genotypes.

## Introduction

Pediatric acute lymphoblastic leukemia (ALL) patients treated according to modern treatment protocols can expect to achieve long-term cure in ~90% of cases, but still a significant proportion of patients suffers from relapse and therapy-related toxicities [1, 2]. Thus, the challenge to further improve treatment for children with ALL addresses not only cure from leukemia, but also reduction of short-term and long-term therapy-associated toxicities [3]. This can be achieved by an optimized potential realization of risk and subsequent treatment adaptation to avoid unwanted events.

The thiopurines 6-mercaptopurine (6-MP) and 6-thioguanine (6-TG) play an essential role in treatment protocols for ALL. Randomized controlled trials (RCTs) comparing the efficacy and toxicity of 6-MP with 6-TG in interim maintenance and maintenance therapy of childhood ALL have demonstrated dose-dependent high rates (>10%) of severe hepatotoxic side effects [4–6]. These side effects have features of sinusoidal obstruction syndrome (SOS) and are associated with long-term exposure to 6-TG. Hepatic SOS after long-term exposure to 6-TG is often reversible, clinical symptoms are commonly less severe, and mortality is very low compared to hepatic SOS in association with hematopoietic stem cell transplantation (HSCT) [6, 7]. Hepatic SOS occurring in association with short-term exposure toward 6-TG has been repeatedly described, but is rare and less well characterized [4, 5, 8–11].

Hepatic SOS, formerly known as veno-occlusive disease (VOD), almost exclusively occurs after exposure to drugs or other toxic stimuli and, in histopathology, correlates with central obstruction of liver sinusoids, liver cell necrosis and hemorrhage [6, 7]. The clinical signs and symptoms of hepatic SOS include abdominal pain and swelling, portal hypertension with liver enzyme elevations and jaundice [6, 7]. A pharmacokinetic study accompanying a British RCT established a role for the thiopurine detoxifying enzyme thiopurine methyltransferase (TPMT) which underlies phenotypically relevant genetic polymorphism [12, 13] in the association of 6-TG with hepatic SOS [14]. However, the latter relationship could not be confirmed by others [8, 15]. As a consequence of the increased toxicity observed with exposure to 6-TG for treatment of ALL, 6-MP remained the standard thiopurine of choice for maintenance treatment of ALL. Since discontinuation of long-term 6-TG application, the previously associated hepatic complications are only rarely observed during treatment for ALL. Nevertheless, due to its anti-leukemic potency, currently the majority of clinical protocols for ALL continue to use short-term applications of 6-TG during intensification elements. Therefore, we analyzed the reported frequencies and characteristics of hepatic SOS in a large multicenter Berlin–Frankfurt–Münster (BFM) RCT on treatment of pediatric ALL in which 6-TG was applied for 2-week intervals during late-intensification therapy.

## Materials/subjects and methods

### Study individuals

**Fig. 1:**
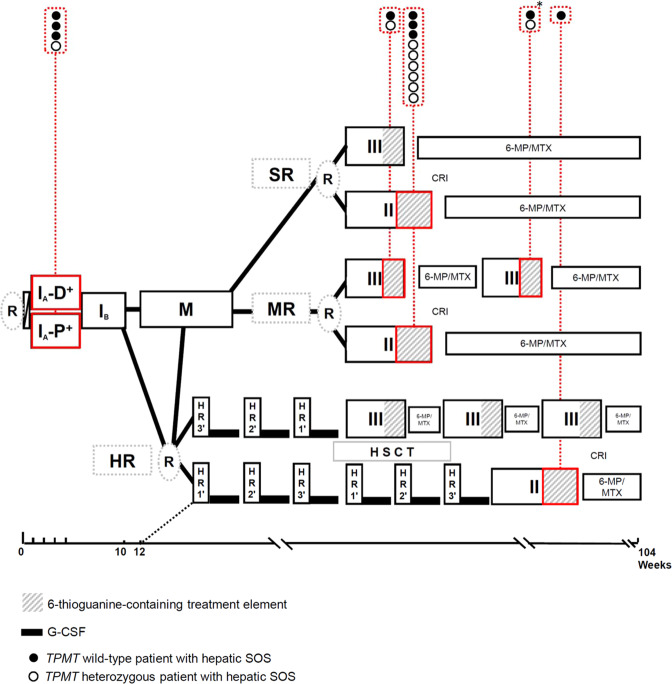
Treatment outline of AIEOP-BFM ALL 2000. Details of treatment elements are listed in Supplementary Table 1. Results of the randomizations (R) and data on allogeneic hematopoietic stem cell transplantation (HSCT) have been published before [16, 27]. SR indicates standard risk; MR, intermediate risk; HR, high risk; closed boxes indicate treatment elements: I_A_, Protocol I phase A; D^+^ indicates induction with dexamethasone and P+ with prednisone; I_B_, Protocol I phase B; M, Protocol M; II, Protocol II; III, Protocol III; HR1′, HR2′ and HR3′ are intensive high-risk treatment blocks; 6-MP/MTX, interim maintenance or maintenance therapy with 6-mercaptopurine and methotrexate; CR cranial irradiation; closed red boxes indicate the treatment elements in which hepatic SOS cases were observed. The asterisk indicates the second episode of hepatic SOS in patient 16 (Table 2) (color figure online).

From August 1, 2000, to May 31, 2010, a total of 3983 ALL patients between 1 and 18 years of age were diagnosed in one of the participating study centers in Germany and registered in trial AIEOP-BFM ALL 2000 [16–18] (Supplementary Fig. 1). Treatment in this trial, which we used as derivation cohort, contained standard multidrug chemotherapeutic regimens (for details see Fig. 1 and Supplementary Table 1) and, in parts of the patient population, cranial irradiation and/or HSCT. 6-TG (60 mg/m^2^/d) was applied for 2 weeks in the second phase of two late-intensification elements, the so-called Protocol II and Protocol III (Fig. 1, Supplementary Table 1). Out of the 3983 patients, 813 had *TPMT* genotype information available (Supplementary Fig. 1). Our replication cohort included 1566 patients between 1 and 18 years of age, diagnosed with pediatric ALL from June 1, 2010, to December 31, 2016, and treated in the non-experimental arms of trial AIEOP-BFM ALL 2009 incorporating the late-intensification element Protocol II [19]. Informed consent was obtained from all patients and/or their guardians. Both study protocols AIEOP-BFM ALL 2000 and AIEOP-BFM ALL 2009 were approved by the competent ethics committees and trials were registered at http://clinicaltrials.gov (NCT00430118 and NCT01117441).

Cases of hepatic SOS were identified through searching the AIEOP-BFM ALL 2000 and AIEOP-BFM ALL 2009 serious adverse event (SAE) databases. An SAE was defined as any untoward medical occurrence that (1) resulted in death; (2) was life threatening (defined as an event in which the patient was at risk of death at the time of the event; it did not refer to an event which hypothetically might have caused death if it would have been more severe); (3) required or prolonged hospitalization; (4) resulted in persistent or significant disability/incapacity; (5) was a congenital anomaly/birth defect in the offspring. Hepatic SOS was characterized according to the Ponte di Legno (PdL) diagnostic criteria for SOS in children with ALL [3] (Supplementary Table 2) and the European Society for Blood and Marrow Transplantation diagnostic criteria for hepatic SOS/VOD in pediatric patients [7]. Patients developing hepatic SOS in association with HSCT were not included in this study.

### *TPMT* genotyping

DNA extraction from fresh mononuclear cells was performed as described previously [20]. Genotyping for *TPMT* was performed by standard RQ-PCR-based genotyping for the variant TPMT*2 and TPMT*3 alleles, which are associated with low TPMT activity [21].

### Statistical analysis

Differences in the distribution of categorical variables were analyzed by chi-squared or Fisher’s exact tests. The McNemar’s test was used on paired dichotomous data. We estimated the odds ratios (derivative cohort) or relative risks (replication cohort) for and calculated their associated 95% confidence intervals [22]. We report exact *p* values. The SPSS (SPSS Inc., Chicago, IL) and SAS (SAS-PC, Version 9.1, Cary, NC: SAS Institute Inc.) statistical packages were used for the analyses.

## Results

We identified 17 patients (0.43%) with hepatic SOS reported as an SAE out of 3983 patients in the derivation cohort (AIEOP-BFM ALL 2000) (see Supplementary Fig. 1 for the analytical workflow of this study). No significant differences with regard to the distribution of important clinical characteristics related to pediatric ALL were detected between patients with reported hepatic SOS in comparison to those without this treatment complication (Table 1). Four patients developed hepatic SOS during induction treatment with a glucocorticoid, vincristine, l-asparaginase, daunorubicin, and intrathecal methotrexate, two of them in association with either acute pancreatitis or severe sepsis (Fig. 1 and Table 2; for treatment details see Supplementary Table 1). In 13 patients, hepatic SOS strongly clustered with short-term exposure to 6-TG during the second phase of late-intensification elements—ten patients were observed in late-intensification Protocol II and three patients during Protocol III (Fig. 1 and Table 2). One patient developed a second episode of hepatic SOS after re-challenge with 6-TG in a second application of Protocol III. The difference of frequencies of hepatic SOS in 6-TG-containing elements compared to other treatment elements was highly significant (*P* ≤ 0.0001). All cases associated with 6-TG exposure were of moderate grade according to PdL criteria [3]. With one exception, all patients received defibrotide (Table 2). All patients with 6-TG-associated hepatic SOS recovered, did not demonstrate residual symptoms such as portal hypertension/splenomegaly or nodular regenerative hyperplasia on follow-up, and are in continuous complete remission (Table 2).

**Table 1:** Characteristics of 17 patients with hepatic sinusoidal obstruction syndrome (SOS) reported as a severe adverse event during treatment for acute lymphoblastic leukemia (ALL) in comparison to the remaining patient population of trial AIEOP-BFM ALL 2000.

	Patients with reported hepatic SOS (*n* = 17) *n* (%)	Patients without reported hepatic SOS (*n* = 3966) *n* (%)	^a^*P*
Gender			
Male	12 (70.6)	2193 (55.3)	
Female	5 (29.4)	1773 (44.7)	0.231
Age at diagnosis (years)			
<10	14 (82.4)	2946 (74.3)	
≥10	3 (17.6)	1020 (25.7)	0.584
Initial WBC^b^ (10^9^/l)			
<50	13 (76.5)	3139 (79.2)	
≥50	4 (23.5)	825 (20.8)	0.766
n.a.	-	2 (0.1)	
Immunophenotype			
B-cell precursor	17 (100)	3305 (85.2)	
T-cell precursor	–	555 (14.3)	
other	–	19 (0.5)	0.223
n.a.	–	87 (2.2)	
CNS disease^c^			
No	15 (88.2)	3584 (90.4)	
Yes	1 (5.9)	135 (3.4)	0.448
n.a.	1 (5.9)	247 (6.2)	
*ETV6-RUNX1* rearrangement			
Negative	13 (76.5)	2883 (72.7)	
Positive	4 (23.5)	839 (21.2)	0.999
n.a.	–	244 (6.2)	
Prednisone response^d^			
Good	16 (94.1)	3539 (89.2)	
Poor	1 (5.9)	394 (9.9)	0.999
n.a.	–	33 (0.8)	
Risk group^e^			
SR	6 (35.3)	1305 (32.9)	
MR	8 (47.1)	2050 (51.7)	
HR	3 (17.6)	609 (15.4)	
Other	–	2 (0.1)	0.845
*TPMT*			
wild-type	8 (35.3)	755 (19.0)	
Heterozygous	9 (47.1)	54 (1.4)	
Deficient	–	4 (0.1)	<0.0001
n.a.	–	3153 (79.5)	

^a^*Pχ*2 or Fisher’s exact test.

^b^*WBC* white blood cell count.

^c^CNS positive: puncture nontraumatic, >5 WBC/μL cerebrospinal fluid with identifiable blasts

^d^Good: <1000 leukemic blood blasts/µl on treatment day 8, poor: ≥1000/µl.

^e^Risk group stratification included minimal residual disease (MRD) analysis and required two MRD targets with sensitivities of ≤1 × 10^−4^, SR patients were MRD-negative on treatment days 33 and 78, HR patients had residual disease of ≥5 × 10^−4^ on treatment day 78, all the remaining MRD results were stratified into the MR group, further HR criteria were prednisone poor response or ≥5% leukemic blasts in the bone marrow on day 33 or positivity for *t*(4;11) or its molecular equivalent (MLL-AF4 gene fusion); were stratified into the high-risk group independent of their MRD results.

**Table 2:** Characteristics of 17 patients with hepatic sinusoidal obstruction syndrome (SOS) during treatment for acute lymphoblastic leukemia (ALL) on trial AIEOP-BFM ALL 2000.

	Treatment phase																
	Induction				Late intensification												
	Protocol I phase A^a^ (Protocol I days 1–33)				Protocol II phase B^b^ (Protocol II days 36–49)										Protocol III phase B^b^ (Protocol III days 15–28)		
Patient	1	2	3	4	5	6	7	8	9	10	11	12	13	14	15	16 ^c^	17
Gender	M	M	F	M	F	M	M	F	F	F	M	M	M	M	M	M	M
Age at diagnosis of ALL (years)	3.4	4.7	14.1	5.9	3.8	13.3	8.6	4.1	4.9	4.6	14.9	2.0	3.0	4.8	3.4	1.1	3.8
SOS diagnosis day from protocol start	31	22	33	33	49	46	52	55	51	54	69	48	54	49	25	29/24	32
Maximum bilirubin (µmol/l)	81	91	275	29	n.a.	131	45	37	49	34	43	32	32	20	83	60/52	41
Maximal weight gain (%)	3.5	n.a.^d^	2.8	14.1	20.7	0.4	10.9	15.9	n.a.	n.a.	3.1	25.2	11.0	23.3	16.7	23.5/n.a.	26.1
Ascites^e^	+	+	n.a.	+	+	+	+	+	+	+	+	+	+	+	+	+/+	+
Tender hepatomegaly	+	+	n.a.	+	+	+	+	+	+	+	+	+	+	+	+	+/+	+
Treatment and clinical course of hepatic SOS^f^	DEF, concomitant pancreatitis, recovered	DEF, concomitant sepsis, death	DEF, recovered	DEF, recovered	DEF, recovered	DEF, recovered	HEP, recovered	DEF, HEP, PR-C, recovered	DEF, recovered	DEF, LMW HEP, recovered	DEF, recovered	DEF, recovered	DEF, HEP, recovered	DEF, HEP, recovered	DEF, recovered	DEF, recovered	DEF, recovered
Risk group for ALL treatment^g^	HR	MR	HR	SR	SR	MR	MR	SR	HR	MR	SR	SR	SR	MR	MR	MR	MR
Final treatment outcome^h^	death in CCR	death before CR	relapse	CCR	CCR	CCR	CCR	CCR	CCR	CCR	CCR	CCR	CCR	CCR	CCR	CCR	CCR
*TPMT* genotype	*1/*1	*1/*3A	*1/*1	*1/*1	*1/*1	*1/*3A	*1/*3A	*1/*3A	*1/*1	*1/*1	*1/*1	*1/*3A	*1/*3A	*1/*3A	*1/*3A	*1/*1	*1/*3A

^a^Seven-day prednisone prephase and induction treatment with prednisone or dexamethasone, vincristine, daunorubicine, l-asparaginase, and intrathecal methotrexate; for exact doses and scheduling see Supplementary Table 1.

^b^Intensification treatment with cyclophosphamide, cytarabine, 6-thioguanine, and intrathecal methotrexate; for exact doses and scheduling see Supplementary Table 1.

^c^Patient with a second episode of hepatic SOS after re-challenge with 6-thioguanine in the second application of Protocol III.

^d^Not available.

^e^ Proven by abdominal ultrasound.

^f^*DEF* Defibrotide, *HEP* heparin, *Pr-C* protein C, *LMW HEP* low-molecular-weight heparin.

^g^Risk group stratification (standard risk, SR; intermediate risk, IR; high risk, HR) was mainly based on minimal residual disease (MRD) analysis after induction (time point [TP] 1) and consolidation therapy (TP2). SR patients were MRD-negative on both TP and HR patients had MRD levels of ≥10^−3^ at TP2. MRD-IR patients had positive MRD detection at either one or both time points but at a level of <10^−3^ at TP2. Patients with prednisone poor-response (PR; ≥ 1000 leukemic blood blasts / µl on treatment day 8) or induction failure (≥5% leukemic blasts in the bone marrow, BM) or positivity for translocations t(9;22) or t(4;11) or their molecular equivalents (BCR/ABL1 or MLL/AF4 rearrangement) were stratified into the HR group independent of their MRD results.

^h^*CR* complete remission (<5% leukemic blasts in the bone marrow), *CCR* continuous complete remission.

The numbers of patients exposed to 6-TG in trial AIEOP-BFM ALL 2000 were 2531 for Protocol II and 1212 for Protocol III. This results in a rate of hepatic SOS in association with 6-TG of 0.40% in Protocol II compared to 0.25% in Protocol III (*P* = 0.47). Co-medication with 6-TG in these two late-intensification elements consisted of similar applications of cytarabine and intrathecal methotrexate, while a single cyclophosphamide application was given at 1000 mg/m^2^ in Protocol II compared to 500 mg/m^2^ in Protocol III (Supplementary Table 1). Thus, we could not evaluate if cyclophosphamide modifies the risk of hepatic SOS in association with 6-TG treatment.

Next, we compared a representative subset of 813 AIEOP-BFM ALL 2000 patients [20] with available *TPMT* genotype data and no reported history of hepatic SOS to the 13 patients with hepatic SOS under exposure to 6-TG during late-intensification treatment (Protocol II/Protocol III). Eight (61.5%) of the 13 patients with hepatic SOS were heterozygous for *TPMT* variants conferring low enzyme activity compared to 54 (6.6%) in the cohort without reported hepatic SOS (Table 1 and Fig. 1). In contrast, only one out the four patients with hepatic SOS during induction treatment carried a low-activity *TPMT* allele (Table 2). The odds ratio for hepatic SOS under exposure to 6-TG was 22.4 (95% confidence interval 7.1–70.7; *P* ≤ 0.0001) for *TPMT* heterozygotes in comparison to *TPMT* wild-type patients. To replicate this association, we analyzed 1566 patients treated on the non-experimental arm of trial AIEOP-BFM ALL 2009. These patients received the same 6-TG containing Protocol II as given in the previous trial AIEOP-BFM ALL 2000. A search of the AIEOP-BFM ALL 2009 SAE database for study individuals with reported hepatic SOS associated with Protocoll II identified nine out of 1566 ALL patients (0.57%) (Supplementary Table 3). Three of the nine patients were heterozygous for *TPMT* alleles conferring low enzyme activity (all TPMT*1/*3A) resulting in a 6.73-fold increased relative risk (95% confidence interval 1.71–26.53; *P* = 0.007) of hepatic SOS in association with 6-TG for heterozygotes in comparison to *TPMT* wild-type patients.

## Discussion

In the present analysis, short-term exposure to 6-TG during treatment for pediatric ALL was associated with hepatic SOS and patients with *TPMT* genotypes conferring low enzyme activity were at increased risk. Supporting the specificity of the association of hepatic SOS with 6-TG exposure, no patient with hepatic SOS in association with the second phase of Protocol I was detected. This treatment element equals the second phases of Protocol II and Protocol III except that 6-MP is applied instead of 6-TG (Supplementary Table 1). Therefore, in a similar treatment context, hepatic SOS recorded as an SAE was only observed in context with application of 6-TG, but virtually absent with 6-MP. The latter observation suggests that replacement of 6-TG by 6-MP in late-intensification elements may abolish or at least significantly reduce the appearance of hepatic SOS during late-intensification. Due to the currently observed good long-term outcome of our patients with hepatic SOS in association with short-term 6-TG and the lack of data on equivalent anti-leukemic efficacy of 6-MP versus 6-TG in late-intensification elements we did not yet change the drug compositions of Protocols II and III. However, we alert clinicians to the specific risk of hepatic SOS under exposure to 6-TG in our clinical protocols. A potential explanation for the differential impact of 6-TG and 6-MP on risk of hepatic SOS may be specific angioprotective properties attributed to 6-MP. In different vascular disease model systems, 6-MP was shown to impact on the expression of adhesion molecules, to reduce monocyte attraction/adhesion, to induce monocyte apoptosis, to inhibit re-stenosis and to attenuate inflammation, while stimulating endothelial coverage [23–26].

In a single institution study, McAtee et al. recently published a series of ten (1.5%) hepatic SOS out of 680 pediatric ALL patients treated at Texas Children’s Cancer Center [9]. Compared to long-term exposure during 6-TG-based maintenance treatment of ALL, hepatic SOS in context of short-term 6-TG exposure was associated with earlier onset and higher mortality (20%) which led the authors to conclude that hepatic SOS after short-term 6-TG treatment may be a clinically distinct phenotype with similarities to hepatic SOS in association with HSCT. Regarding timing of hepatic SOS our data confirm the observation of McAtee et al. of an early onset in close association with 6-TG application (Table 2) [9]. Similarly, we also observed a high frequency of low *TPMT* activity in our patients. This may support the hypothesis that altered 6-TG metabolism may either modify the severity of hepatic SOS or—as associations with long-term exposure are contradictory—reflect a stronger effect of variant *TPMT* in association with short-term exposure during late intensification. In addition, our data provide a further instructive example for the importance to subject pediatric ALL patients treated with thiopurines to *TPMT* genotyping. Intriguingly, McAtee et al. described confirmed or suspected infections prior to diagnosis of hepatic SOS in six out of ten cases with hepatic SOS [9]. Due to unavailability of data, we cannot reliably evaluate this association in our study. The single patient in our study suffering from concomitant sepsis who finally died in association with hepatic SOS was observed during induction treatment and not in association with 6-TG. Although also potential differences in protocol-specific treatment exposures may have contributed here, the lower frequency of hepatic SOS in our study compared to McAtee’s study is likely due to the inclusion of hepatic SOS reported as an SAE, only. This indicates that we probably did not capture all/less severe episodes of hepatic SOS. Therefore, careful monitoring for signs of hepatic SOS in association with short-term 6-TG exposure in future studies will have to characterize the entire clinical spectrum and determine the true incidence of this complication.

We conclude that hepatic SOS reported as an SAE on contemporary ALL-BFM trials cluster with short-term application of 6-TG during late-intensification elements and are associated with *TPMT* genotype. Particularly for *TPMT* heterozygotes, replacement of 6-TG by 6-MP may be an effective measure to reduce the incidence of hepatic SOS during treatment for pediatric ALL.

## Supplementary information

Supplemental Material
