# Supplementary material for: Hepatic sinusoidal obstruction syndrome and short-term application of 6-thioguanine in pediatric acute lymphoblastic leukemia
Source: Leukemia. 2021 Mar 13;35(9):2650–7. doi: 10.1038/s41375-021-01203-7 (PMC8410596; doi:10.1038/s41375-021-01203-7)

Supplementary table 1. Treatment details of protocol AIEOP-BFM ALL 2000.

Treatment phase/drug[a]	Single or daily dose	Days of application per phase[a]
<u>Prephase</u><br>Prednisone (PO/IV)<br>Methotrexate (IT)	 60 mg/m^2/d<br>12 mg/dose[b]	 1-7<br>1
<u>Induction</u><br>*Protocol IA*<br>Prednisone/Prednisolone (PO/IV) *or*[c]<br>Dexamethasone (PO/IV)<br>Vincristine (IV)<br>Daunorubicin (PI over 1 h)<br>L-Asparaginase (PI over 1 h)<br>Methotrexate (IT)	 <br>60 mg/m^2/d<br>10 mg/m^2/d<br>1.5 mg/m^2/dose (max 2 mg)<br>30 mg/m^2/dose<br>5000 IU/m^2/dose<br>12 mg/dose[b]	 <br>8-28[d]<br>8-28[d]<br>8, 15, 22, 29<br>8, 15, 22, 29<br>12, 15, 18, 21, 24, 27, 30, 33<br>12, 33[e]
<u>Consolidation</u><br>*Protocol IB*<br>Cyclophosphamide (PI over 1 h)<br>Cytarabine (IV)<br>6-Mercaptopurine (PO)<br>Methotrexate (IT)	 <br>1000 mg/m^2/dose<br>75 mg/m^2/dose<br>60 mg/m^2/d<br>12 mg/dose[b]	 <br>36, 64<br>38-41, 45-48, 52-55, 59-62<br>36-63<br>45, 59
<u>Extra-Compartment Therapy</u><br><u>(only SR/MR)</u><br>*Protocol M:*<br>6-Mercaptopurine (PO)<br>Methotrexate (PI over 24 h)[f]<br>Methotrexate (IT)	 <br><br>25 mg/m^2/d<br>5000 mg/m^2/dose<br>12 mg/dose[b]	 <br><br>1-56<br>8, 22, 36, 50<br>8, 22, 36, 50
<u>Intensive Consolidation (only HR)</u><br>*Element HR-1'*<br>Dexamethasone (PO/IV)<br>Vincristine (IV)<br>Methotrexate (PI over 24 h)[f]<br>Cyclophosphamide (PI over 1 h)<br>Cytarabine (PI over 3 h)<br>L-Asparaginase (PI over 2 h)<br>Methotrexate/Cytarabine/<br>Prednisolone (IT)	 20 mg/m^2/d<br>1.5 mg/m^2 (max 2 mg)<br>5000 mg/m^2/dose<br>200 mg/m^2/dose<br>2 g/m^2/dose<br>25,000 IU/m^2/dose<br>12/30/10 mg/dose[b]	 1-5<br>1, 6<br>1<br>2-4 (5 doses, 12 h intervals)<br>5 (2 doses, 12 h interval)<br>6, 11<br>1
Element HR-2'<br>Dexamethasone (PO/IV)<br>Vindesine (IV)<br>Methotrexate (PI over 24 h)[f]<br>Ifosfamide (PI over 1 h)<br>Daunorubicin (PI over 24 h)<br>L-Asparaginase (PI over 2 h)<br>Methotrexate/Cytarabine/<br>Prednisolone (IT)	 20 mg/m^2/d<br>3 mg/m^2/dose (max 5 mg)<br>5000 mg/m^2/dose<br>800 mg/m^2/dose<br>30 mg/m^2/dose<br>25,000 IU/m^2/dose<br>12/30/10 mg/dose[b]	 1-5<br>1, 6<br>1<br>2-4 (5 doses, 12 h intervals)<br>5<br>6, 11<br>1[g]

Treatment phase/drug[a]	Single or daily dose	Days of application per phase[a]
Element HR-3'		
Dexamethasone (PO/IV)	20 mg/m^2/d	1-5
Cytarabine (PI over 3 h)	2 g/m^2/dose	1-2 (4 doses, 12 h intervals)
Etoposide (PI over 1 h)	100 mg/m^2/dose	3-5 (5 doses, 12 h intervals)
L-Asparaginase (PI over 2 h)	25,000 IU/m^2/dose	6, 11
Methotrexate/Cytarabine/ Prednisolone (IT)	12/30/10 mg/dose[b]	5
Reinduction		
Protocol II		
Dexamethasone (PO/IV)	10 mg/m^2/d	1-21[d]
Vincristine (IV)	1.5 mg/m^2/dose (max 2 mg)	8, 15, 22, 29
Doxorubicin (PI over 1 h)	30 mg/m^2/dose	8, 15, 22, 29
L-Asparaginase (PI over 1 h)	10,000 IU/m^2/dose	8, 11, 15, 18
Cyclophosphamide (PI over 1 h)	1000 mg/m^2/dose	36
Cytarabine (IV)	75 mg/m^2/dose	38-41, 45-48
6-Thioguanine (PO)	60 mg/m^2/d	36-49
Methotrexate (IT)	12 mg/dose[b]	45, 59[g]
Protocol III		
Dexamethasone (PO)	10 mg/m^2/d	1-14[d]
Vincristine (IV)	1.5 mg/m^2/dose (max 2 mg)	1, 8
Doxorubicin (PI over 1 h)	30 mg/m^2/dose	1, 8
L-Asparaginase (PI over 1 h)	10,000 IU/m^2/dose	1, 4, 8, 11
Cyclophosphamide (PI over 1 h)	500 mg/m^2/dose	15
Cytarabine (IV)	75 mg/m^2/dose	17-20, 24-27
6-Thioguanine (PO)	60 mg/m^2/d	15-28
Methotrexate (IT)	12 mg/dose[b]	17, 24[g]
Interim Maintenance		
Methotrexate (PO)	20 mg/m^2/dose[h]	once a week
6-Mercaptopurine (PO)	50 mg/m^2/d[i]	daily
Maintenance[I]		
Methotrexate (PO)	20 mg/m^2/dose[h]	once a week
6-Mercaptopurine (PO)	50 mg/m^2/d[i]	daily
Cranial irradiadion	12 Gy/18 Gy/24 Gy	

[a] PO indicates orally; IV, intravenous push; PI, intravenous infusion; IT, intrathecally; adjustments of time schedule were allowed if clinical condition and bone marrow recovery were inadequate

[b] Doses of IT drugs were adjusted for children <3 years of age

[c] Randomization

[d] Steroids were tapered over 9 additional days

[e] Additional IT therapy on day 18 and 27 was administered to patients with CNS status CNS3 and CNS2 or TLP+

[f] A loading dose of 10% was infused over 30 min, the remaining 90% over 23.5 h. Leucovorin rescue was given at hour 42, 48, and 54 (each 15 mg/m^2). Doses of leucovorin rescue were adjusted, if MTX levels were > 1.0 µmol/l at hour 42 or later. If the MTX level at hour 54 was > 0.25 µmol/l, rescue was continued at six-hour intervals until MTX levels were ≤ 0.25 µmol/l.

[g] Patients with CNS status CNS 3 received additional IT therapy on day 5 in element HR-2', on day 1 and 18 in Protocol II and on day 1 in Protocol III

[h] Doses were adjusted to WBC (target range 2.0-3.0 x10^9/L)

[I] Maintenance was given from the end of intensive chemotherapy until 104 weeks after diagnosis

Supplementary table 2. Definition of hepatic sinusoidal obstruction syndrome according to the toxicity working group of the Ponte di Legno consortium.

Fulfilment of at least three of five, otherwise unexplained, criteria*:	
- Hepatomegaly	
- Hyperbilirubinaemia more than UNL	
- Ascites	
- Weight gain of at least 5%	
- Thrombocytopenia (transfusion-resistant and/or otherwise unexplained by treatment)	
Grading:	
- Mild:	bilirubin less than 103 µmol/L and weight gain less than 5%
- Moderate:	bilirubin 103–342 µmol/L and/or weight gain more than 5% or ascites
- Severe:	bilirubin more than 342 µmol/L and/or respiratory or renal failure or hepatic encephalopathy
- Death due to sinusoidal syndrome	

***Doppler ultrasound could document changes in hepatic portal venous flow and rule out alternative causes, but normal findings do not exclude sinusoidal obstruction syndrome

Supplementary table 3. Comparison of characteristics of 1566 patients with and without hepatic sinusoidal obstruction syndrome (SOS) reported as severe adverse event during treatment of acute lymphoblastic leukemia (ALL) on the non-interventional arms of trial AIEOP-BFM ALL 2009.

	**Patients with reported hepatic SOS (n = 9)**<br><br>**n (%)**	**Patients without reported hepatic SOS (n = 1557)**<br><br>**n (%)**	[e]***P***
Gender			
Male	5 (55.6)	889 (57.1)	
Female	4 (44.4)	668 (42.9)	0.999
Age at diagnosis (years)			
<10	8 (88.9)	1213 (77.9)	
≥10	1 (11.1)	344 (22.1)	0.693
Initial WBC[a] (10^9/l)			
<50	7 (77.8)	1320 (84.8)	
≥50	2 (22.2)	236 (15.2)	0.634
n.a.	-	1 (0.1)	
Immunophenotype			
B cell precursor	8 (88.9)	1380 (88.6)	
T cell precursor	1 (11.1)	173 (11.1)	
other	-	-	0.999
n.a.	-	4 (0.3)	
CNS disease[b]			
no	8 (88.9)	1454 (93.4)	
yes	1 (11.1)	31 (2.0)	0.178
n.a.	-	72 (4.6)	
ETV6-RUNX1 rearrangement			
negative	7 (77.8)	1102 (70.8)	
positive	2 (22.2)	446 (28.6)	0.999
n.a.	-	9 (0.6)	
Prednisone response[c]			
good	9 (100)	1547 (99.4)	
poor	-	-	0.999
n.a.	-	10 (0.6)	
Risk group[d]			
SR	4 (44.4)	800 (51.4)	
MR	4 (44.4)	677 (43.5)	0.999
HR	-	-	
other	1 (11.1)	80 (5.1)	

TPMT			
wildtype	6 (66.7)	1447 (19.0)	
heterozygous	3 (33.3)	105 (6.7)	0.020
deficient	-	5 (0.3)	

[a]WBC: white blood cell count
[b]CNS positive: puncture nontraumatic, >5 WBC/µL cerebrospinal fluid with identifiable blasts
[c]Good: <1000 leukemic blood blasts/µl on treatment day 8, poor: ≥1000/µl
[d]Risk group stratification included DNA-based minimal residual disease (MRD) analysis, SR patients were MRD-negative on treatment days 33 and 78, HR patients had residual disease of $\geq 5x10^{-4}$ on treatment day 78 or slow early response (SER, only applicable to B cell precursor ALL) as defined by MRD of $\geq 10^{-3}$ on day 33 and positivity on day 78, all the remaining MRD results were stratified into the MR group, further HR criteria were prednisone poor-response, flow cytometry-based MRD on day 15 of ≥10 % lymphoblasts in bthe one marrow or ≥5% leukemic blasts in the bone marrow by cytomorphological assessment on day 33, positivity for t(4;11) or its molecular equivalent (*MLL-AF4* gene fusion), or hypodiploidy (defined by < 45 chromosomes and/or DNA index of < 0.8) were stratified into the high-risk group independent of their MRD results
[e]*P* Fisher's exact test
[f]Deficient patients were treated by therapeutic drug-monitoring and excluded from risk analyses

Supplementary figure 1. Overview of included patients and performed analyses in the derivation and replication cohorts.

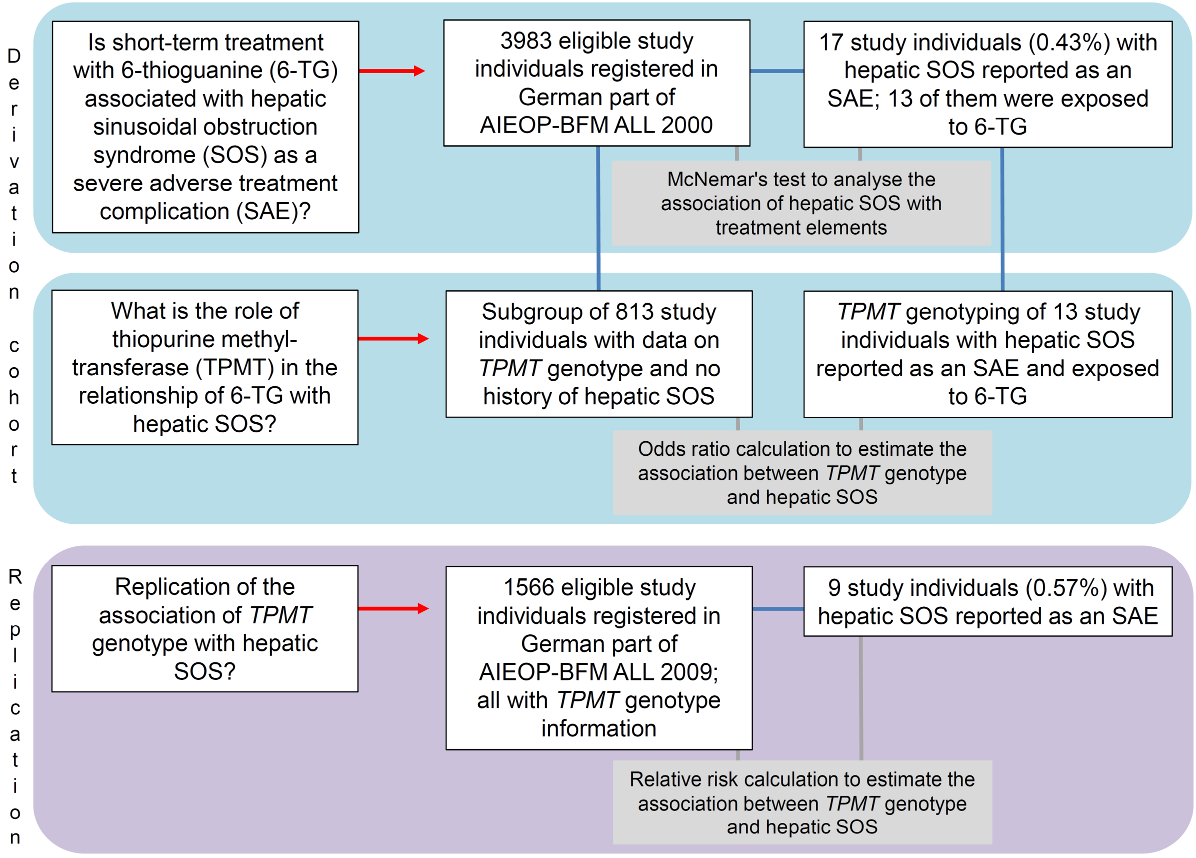

Supplement: Supplementary file 1 — Supplemental Material [file 41375_2021_1203_MOESM1_ESM.pdf]
